# Single shot zonal oblique multislice SE-EPI diffusion-weighted imaging with low to ultra-high b-values for the differentiation of benign and malignant vertebral spinal fractures

**DOI:** 10.1016/j.ejro.2021.100377

**Published:** 2021-09-22

**Authors:** Elisabeth Sartoretti, Sabine Sartoretti-Schefer, Luuk van Smoorenburg, Barbara Eichenberger, Árpád Schwenk, David Czell, Alex Alfieri, Andreas Gutzeit, Manoj Mannil, Christoph A. Binkert, Michael Wyss, Thomas Sartoretti

**Affiliations:** a Institute of Radiology, Kantonsspital Winterthur, Brauerstrasse 15, 8401, Winterthur, Switzerland; b Faculty of Medicine, University of Zürich, Zürich, Switzerland; c Department of Neurosurgery, Kantonsspital Winterthur, Winterthur, Switzerland; d Department of Radiology, Paracelsus Medical University, Strubergasse 21, 5020, Salzburg, Austria; e Clinic of Radiology, University Hospital Münster, WWU University of Münster, Münster, Germany; f Philips Healthsystems, Zürich, Switzerland; g Department of Radiology and Nuclear Medicine, Maastricht University Medical Center, Maastricht University, Maastricht, the Netherlands

**Keywords:** MRI, Magnetic Resonance Imaging, DWI, Diffusion Weighted Imaging, ADC, Apparent Diffusion Coefficient, SShot, Single Shot, MShot, Multi Shot, SE-EPI, Spin Echo – Echo Planar Imaging, FOV, Field of View, ZOOM, Zonal Oblique Multislice, STIR, Short Tau Inversion Recovery, PET-CT, Positron Emission Tomography – Computed Tomography, DXA, Dual Energy X-Ray Absorptiometry, T1w, T1-weighted, T2w, T2-weighted, TSE, Turbo Spin Echo, SI, Signal Intensity, SIR, Signal Intensity Ratio, AUC, Area Under the Curve, ROC, Receiver Operating Characteristics, Diffusion magnetic resonance imaging, Spinal fractures, Vertebral body, Magnetic resonance imaging

## Abstract

**Purpose:**

To investigate the diagnostic yield of low to ultra-high b-values for the differentiation of benign from malignant vertebral fractures using a state-of-the-art single-shot zonal-oblique-multislice spin-echo echo-planar diffusion-weighted imaging sequence (SShot ZOOM SE-EPI DWI).

**Materials and Methods:**

66 patients (34 malignant, 32 benign) were examined on 1.5 T MR scanners. ADC maps were generated from b-values of 0,400; 0,1000 and 0,2000s/mm^2^. ROIs were placed into the fracture of interest on ADC maps and trace images and into adjacent normal vertebral bodies on trace images. The ADC of fractures and the Signal-Intensity-Ratio (SIR) of fractures relative to normal vertebral bodies on trace images were considered quantitative metrics. The appearance of the fracture of interest was graded qualitatively as iso-, hypo-, or hyperintense relative to normal vertebrae.

**Results:**

ADC achieved an area under the curve (AUC) of 0.785/0.698/0.592 for b = 0,400/0,1000/0,2000s/mm^2^ ADC maps respectively. SIR achieved an AUC of 0.841/0.919/0.917 for b = 400/1000/2000s/mm^2^ trace images respectively. In qualitative analyses, only b = 2000s/mm^2^ trace images were diagnostically valuable (sensitivity:1, specificity:0.794). Machine learning models incorporating all qualitative and quantitative metrics achieved an AUC of 0.95/0.98/0.98 for b-values of 400/1000/2000s/mm^2^ respectively. The model incorporating only qualitative metrics from b = 2000s/mm^2^ achieved an AUC of 0.97.

**Conclusion:**

By using quantitative and qualitative metrics from SShot ZOOM SE-EPI DWI, benign and malignant vertebral fractures can be differentiated with high diagnostic accuracy. Importantly qualitative analysis of ultra-high b-value images may suffice for differentiation as well.

## 1 Introduction

Both benign osteoporotic compression fractures as well as pathologic metastatic vertebral fractures due to vertebral metastases are common conditions, especially in elderly patients.

In clinical routine, radiologists have to differentiate these two entities as this paves the subsequent therapeutic process.

However, the differentiation can be challenging on conventional MR images as both entities may result in similar changes in signal intensity on pre- and postcontrast T1-weighted (w) and on T2w images [[1], [2], [3], [4], [5], [6]].

Recent endeavors in improving the differentiation of benign and malignant vertebral fractures with MRI have focused on diffusion-weighted imaging (DWI). Investigators have thereby proposed both qualitative and quantitative approaches based on trace images and apparent diffusion coefficient (ADC) maps in an effort to improve the differentiation of these entities [5,7].

Interestingly, all these studies used DWI protocols with low to moderate b-values. Specifically, b-values of around 400 s/mm^2^ were used most frequently with a single study using a high b-value of 1400 s/mm^2^ [1,5,7]. Furthermore, previous studies have employed single shot (SShot) or multi shot (MShot) spin-echo echo-planar-imaging (SE-EPI) sequences [1]. For spine imaging, these DWI sequences are suboptimal as they are susceptible to off-resonance artifacts created by magnetic field inhomogeneity surrounding the spinal column and spinal cord. Additionally, given the small voxel sizes required to accurately image the spine and the large field of view (FOV) required to avoid aliasing of tissues outside the FOV, these artifacts intensify even more.

Recently, novel MRI sequences have been developed to counteract these technical limitations. One promising method is the zonal oblique multislice (ZOOM)-EPI technique. This technique allows for imaging with reduced FOV without aliasing, thus also reducing image blurring and geometrical distortion [8].

Importantly, with this sequence, ultra-high b-values can be acquired robustly at 1.5 T at an acceptable scan time. Ultra-high b-values provide better imaging contrast, greater tissue diffusivity and less T2 shine-through effect than lower b-values [9,10].

Accordingly, in brain and body imaging, ultra-high b-values have shown promise in a clinical setting. Specifically, it has been suggested that diagnostic performance can be improved by using ultra-high b-values as compared to lower b-values [10,11].

Given these considerations we sought to assess the diagnostic yield of low to ultra-high b-values for the differentiation of benign from malignant vertebral fractures using a single-shot zonal oblique multislice spin-echo echo-planar diffusion-weighted imaging sequence (SShot ZOOM SE-EPI DWI) at 1.5 T. Importantly, we hypothesized that ultra-high b-values acquired with a state-of-the-art optimized DWI sequence may further improve the capability of DWI for the differentiation of these two entities.

To this extent, using a SShot ZOOM SE-EPI DWI sequence, we acquired low (b = 400 s/mm^2^), high (b = 1000 s/mm^2^) and ultra-high (b = 2000 s/mm^2^) b-values in a representative patient cohort and compared the diagnostic performance of qualitative and quantitative metrics derived from ADC maps and DWI trace images to differentiate benign from malignant vertebral fractures.

## 2 Materials and methods

### 2.1 Study subjects

In this institutional review board approved, head-to-head, intra-individual comparison study 66 patients were enrolled. Patients underwent spine MR imaging using a dedicated protocol between January and June 2020. In line with previous studies [3], we enrolled patients referred for MRI examination due to suspicion of acute benign (osteoporotic) vertebral body fractures (25 females, 7 males, mean age 73.9 years with range 56–90 years) and acute malignant (metastatic) vertebral body fractures (9 females, 25 males, mean age 76.4 years with range 48–77 years). Inclusion criteria for all patients were as follows: 18 years or older, back pain at the level of the vertebral fracture, presence of bone marrow edema at the level of the fracture as assessed on Short Tau Inversion Recovery (STIR). Exclusion criteria were as follows: Pregnancy, contraindications to MRI, hematological disorders. Patients were allocated to the benign or malignant group based on clinical follow-up combined with information gained from histology (as obtained during surgery or after CT-guided biopsy), follow-up MRI (appearance of edema, possible morphological signs of malignancy), PET-CT (definite pathologic SUV in case of malignant cause), dual energy x-ray absorptiometry (DXA) and subsequent CT imaging [3]. For patients allocated to the malignant group, metastatic vertebral body fractures were due to prostate carcinoma in 12 patients, breast carcinoma in 9 patients, non-small cell lung cancer (NSCLC) in 9 patients and hepatocellular carcinoma in 4 patients.

### 2.2 MRI

All patients underwent spine MRI on one of two 1.5 T MRI scanners (Philips Achieva (A) and Ingenia (B), Best, the Netherlands) at a single tertiary center. Scanner A was on software release 5.6 with a 5-channel spine coil, scanner B was on software release 5.7 with a 16-channel built-in posterior spine coil. The imaging protocol consists of sagittal T1w TSE, T2w TSE, STIR T2w TSE, diffusion weighted imaging (DWI) and transverse T2w TSE sequences.

The DWI sequence used in this work uses a non-coplanar excitation combined with outer volume suppression. A detailed description of the sequence can be found elsewhere [12]. Specifically this reduced field of view (FOV) imaging technique is referred to as diffusion weighted zonal oblique spin-echo echo-planar imaging (DW ZOOM SE-EPI). The main applications for small-FOV DW imaging are DW imaging of the prostate, spinal cord, pancreas, breast, and heart, where a relatively small area of interest surrounded by tissue of less interest is depicted with high resolution and mainly leads to less image distortion [[12], [13], [14], [15], [16], [17], [18], [19]]. Sequence parameters can be found in Table 1. As suggested and recommended elsewhere [20,21] two-point b-value ADC maps were generated from the following b-value combinations: b = 0,400; b = 0,1000 and b = 0,2000 s/mm^2^ respectively. ADC maps were computed based on a mono-exponential fitting model using the inline ADC calculation tool on the scanner console.

**Table 1:** Sequence parameters.

	SShot ZOOM SE-EPI DWI b0, b400, b1000, b2000
Field of View (FoV)	220 × 100 × 60 mm^3^
Acquired voxel size	2.75 × 2.75 × 5.0 mm^3^
Reconstructed voxel size	1.2 × 1.2 × 5.0 mm^3^
Number of slices	12
Repetition time (TR)	2500 ms
Echo time (TE)	93 ms
Flip angle	90°
EPI factor	47
Number of signal averages (NSA)	b0 = 1, b400 = 2, b1000 = 8, b2000 = 12
Receiver bandwidth	33.3 Hz / pixel
Acquisition time [mm:ss]	05:30

### 2.3 Image analysis

All quantitative analyses were performed twice by two readers (board-certified neuroradiologist with 30 years of experience and trainee with 3 years of experience in imaging) in consensus in a blinded and randomized manner. The averaged values were considered representative for statistical analyses.

Readers were provided with T1w, T2w, STIR T2w, DWI and ADC images/maps. For each patient, the readers selected the fracture with the highest signal intensity on STIR at the level of back pain [3]. As suggested elsewhere, in case of multiple (acute) fractures, only one lesion was considered for further analyses [3]. For a given fracture, ROIs were manually drawn on the area with hyperintense signal on STIR and hypointense signal on T1w images. Then, ROIs were copied to the ADC maps and DWI trace images using the copy-and-paste function. In case of distortions, ROI placement was adjusted manually after copying.

Additionally, for the DWI trace images of each patient, quantitative values for normal vertebral bodies were obtained. To this extent, ROIs were manually drawn on a normal vertebral body on T1w images situated below or above the level of the fracture and were copied to the DWI trace images using the copy-and-paste function. In case of distortions, ROI placement was adjusted manually after copying.

Using the mean values from these ROIs, the following quantitative metrics were derived:

1.) For the ADC maps we used the ADC value derived from the fracture as a biomarker [3,22,23].
2.) In an effort to quantify the appearance of lesions on DWI trace images, we also computed a further metric as proposed previously by Wang et al. [24]. Specifically, the quotient between the mean signal intensity (SI) of the vertebral fracture and the mean SI of the normal vertebral body on DWI trace images was considered as a biomarker (signal intensity ratio – SIR). The formula is as follows:

$$SIR=\;\frac{{Mean\;SI}_{Fracture}\;}{{Mean\;SI}_{Normal\;Vertebral\;Body}}$$

Ultimately, the two readers also performed a qualitative analysis of the images in consensus. Specifically, for each patient they rated the appearance of the fracture of interest on ADC maps and DWI trace images relative to the normal bone marrow of adjacent vertebra and structures as either hypo- iso- or hyperintense [2]. The qualitative appearance of fractures on STIR T2w, T1w and T2w images was also recorded in this manner.

### 2.4 Data analysis

First the diagnostic performance of metrics, stratified by each b-value, was assessed individually. To assess the combined diagnostic performance of all metrics and the diagnostic yield of DWI in general, machine learning (ML) backed models were used.

#### 2.4.1 Statistical analysis for individual metrics

Normality was checked with histograms, boxplots, quantile-quantile-plots and Shapiro-Wilks tests. In case of normally distributed data, student’s t-tests and in case of non-normally distributed data, Mann-Whitney U tests were computed to check for statistical differences between the two study groups. Additionally, receiver operating characteristics (ROC) analyses were performed on the data to quantify the (individual) performance of the various metrics in differentiating benign from malignant vertebral fractures. In this regard, the area under the curve (AUC), and the best cutoff based on the Youden index were computed. Sensitivity and specificity based on the optimal cutoff were also determined. Wherever appropriate, p-values were corrected for multiple comparisons with the Holm method. Adjusted p-values < 0.05 were considered significant. All statistical analyses were performed in the R programming language (version 3.6.3) using the packages “ggplot2”, “rstatix”, and “pROC”.

#### 2.4.2 Machine learning

To quantify the combined performance of all qualitative and quantitative metrics derived from a single b-value and from DWI in general, we used machine learning (ML). We built, trained, and evaluated 9 different models. For each b-value, two models (full, reduced) were designed. In the full model, both qualitative (appearance of fracture on STIR T2w, T1w and T2w images, DWI trace images and ADC maps) and quantitative metrics (ADC and SIR values of fracture) were available as input data (for example, for b = 400 s/mm^2^ there were 5 qualitative and 2 quantitative parameters as input data), and in the reduced model, only the qualitative metrics were available as input data (for example, for b = 400 s/mm^2^ there were only 5 qualitative parameters as input data). As a baseline (i.e. to quantify the overall diagnostic yield of DWI in general), a model was also calculated that included only the (qualitative) image information from conventional STIR T2w, T1w, and T2w imaging.

ML was performed in the R programming language using the package “healthcareai”. In brief, for each model, three algorithms (Random Forest, Extreme Gradient Boosting and Regularized Regression) were individually fitted and optimized iteratively. The data was randomly split in a 90:10 ratio for training and testing respectively. For training, models were tuned via 5-fold cross validation over 10 combinations of hyperparameter values. The optimal algorithm with the optimal hyperparameter values was selected based on the AUC-ROC performance metric. The optimal algorithm was then tested on the final 10 % of data reserved for testing.

## 3 Results

**Fig. 1:**
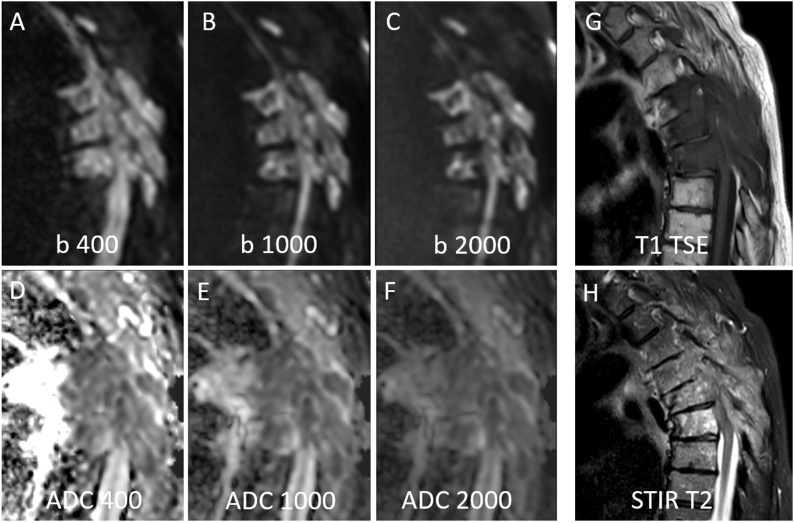
73 years old female patient with multiple malignant (i.e. metastatic) vertebral body fractures of thoracic vertebrae TH4 – TH6. Hyperintense signal of fractures relative to normal vertebral bodies on all DWI images with b400 (Fig. 1A), b1000 (Fig. 1B) and b2000 (Fig. 1C). The signal intensity on ADC decreases with highest values on b = 0,400 ADC images (Fig. 1D), followed by b = 0,1000 ADC images (Fig. 1E) and lowest values on b = 0,2000 ADC images (Fig. 1F). The fracture is hypointense on T1w TSE images (Fig. 1G) and inhomogeneously hyperintense on STIR T2w images (Fig. 1H). For ROI measurements the vertebral body Th6 with the most intense STIR hyperintensity was chosen (Fig. 1H).

**Fig. 2:**
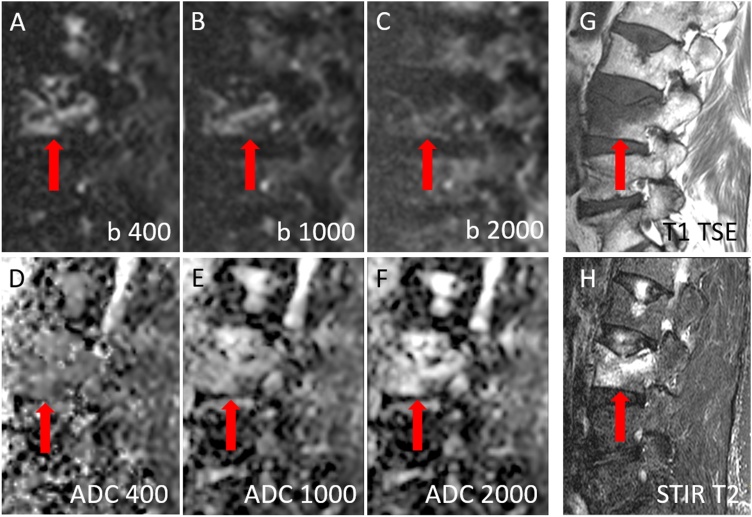
69 years old female patient with osteoporotic vertebral body fracture of lumbar vertebra L3 (red arrow). Hyperintense signal of fractures relative to normal vertebral bodies on DWI with b400 (Fig. 2A) and on DWI with b1000 (Fig. 2B), but no hyperintensity on DWI with b2000 (Fig. 2C). Moderate to high signal intensity on ADC of b = 0,400 (Fig. 2D), on ADC of b = 0,1000 (Fig. 2E) and on ADC of b = 0,2000 images (Fig. 2F). The fracture is hypointense on T1w TSE images (Fig. 2G) and inhomogeneously hyperintense on STIR T2w images (Fig. 2H) (For interpretation of the references to colour in this figure legend, the reader is referred to the web version of this article).

**Fig. 3:**
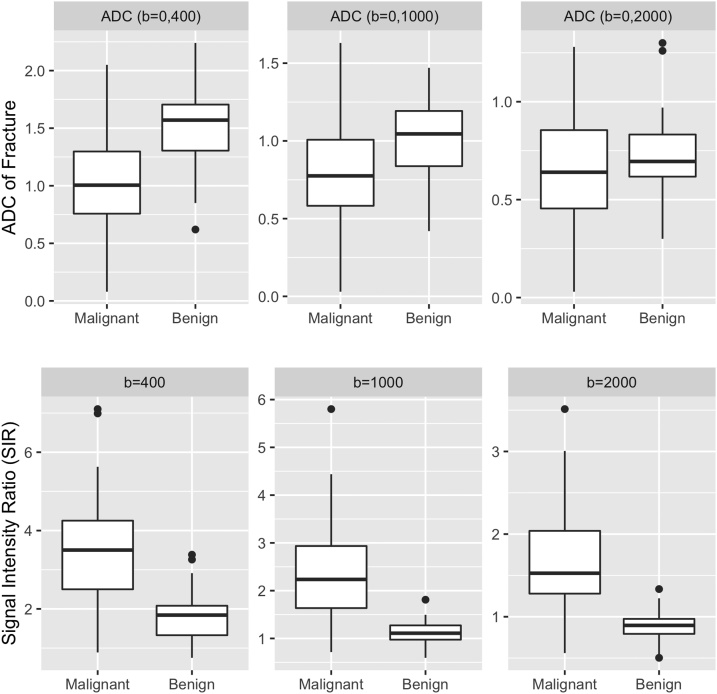
Boxplots depicting quantitative data. The line in the box shows the median, the lower and upper hinges correspond to the first and third quartiles. The upper/lower whisker extends from the hinge to the largest/smallest value no further than 1.5 * IQR from the hinge.

**Fig. 4:**
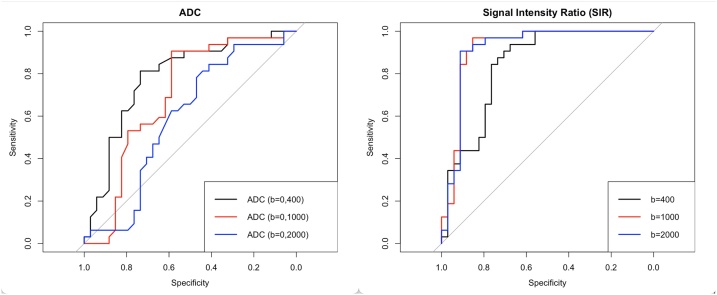
Receiver operating characteristics (ROC) curves for the quantitative metrics ADC and SIR.

**Fig. 5:**
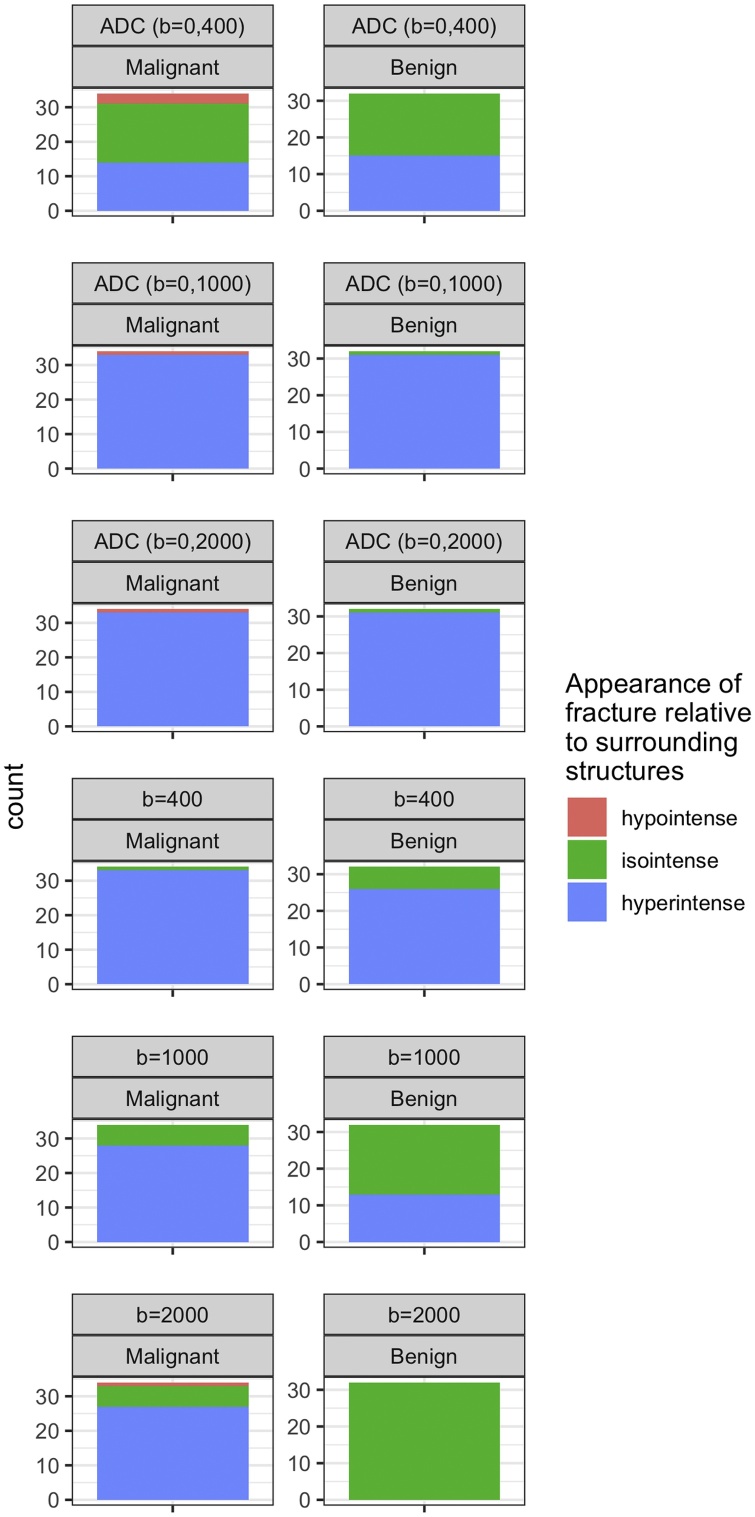
Overview of qualitative data. The frequency of appearances of the fractures of interest are depicted as bar plots.

Typical image examples are shown in Fig. 1, Fig. 2. A detailed overview of the data can be found in Table 2, Table 3 and in Fig. 3, Fig. 4, Fig. 5.

**Table 2:** Overview of quantitative data.

Mean ± Standard Deviation - Median; [Interquartile Range]	ADC (b = 0,400 s/mm^2^) [x 10^–3^ mm^2^/s]	ADC (b = 0, 1000 s/mm^2^) [x 10^–3^ mm^2^/s]	ADC (b = 0, 2000 s/mm^2^) [x 10^–3^ mm^2^/s]	SIR (b = 400 s/mm^2^) [arbitrary units]	SIR (b = 1000 s/mm^2^) [arbitrary units]	SIR (b = 2000 s/mm^2^) [arbitrary units]
Malignant Fracture	1.054 ± 0.454–1.005; [0.758; 1.3]	0.837 ± 0.38−0.775; [0.583; 1.008]	0.663 ± 0.293−0.64; [0.455; 0.855]	3.493 ± 1.481–3.502; [2.501; 4.253]	2.441 ± 1.143–2.235; [1.635; 2.935]	1.69 ± 0.666–1.527; [1.28; 2.039]
Benign Fracture	1.505 ± 0.363–1.57; [1.305; 1.705]	1.033 ± 0.244–1.045; [0.838; 1.193]	0.728 ± 0.218−0.695; [0.618; 0.833]	1.833 ± 0.658–1.842; [1.328; 2.081]	1.101 ± 0.268–1.527; [1.28; 2.039]	0.882 ± 0.179−0.895; [0.793; 0.974]

**Table 3:** : Overview of qualitative data.

	ADC (b = 0,400 s/mm^2^)	ADC (b = 0, 1000 s/mm^2^)	ADC (b = 0, 2000 s/mm^2^)	DWI Trace Image (b = 400 s/mm^2^)	DWI Trace Image (b = 1000 s/mm^2^)	DWI Trace Image (b = 2000 s/mm^2^)
Malignant Fracture (n = 34)	Hypointense: 3	Hypointense: 1	Hypointense: 1	Hypointense: 0	Hypointense: 0	Hypointense: 1
	Isointense: 17	Isointense: 0	Isointense: 0	Isointense: 1	Isointense: 6	Isointense: 6
	Hyperintense: 14	Hyperintense: 33	Hyperintense: 33	Hyperintense: 33	Hyperintense: 28	Hyperintense: 27
Benign Fracture (n = 32)	Hypointense: 0	Hypointense: 0	Hypointense: 0	Hypointense: 0	Hypointense: 0	Hypointense: 0
	Isointense: 17	Isointense: 1	Isointense: 1	Isointense: 6	Isointense: 19	Isointense: 32
	Hyperintense: 15	Hyperintense: 31	Hyperintense: 31	Hyperintense: 26	Hyperintense: 13	Hyperintense: 0

### 3.1 Performance of individual metrics

#### 3.1.1 ADC

In brief, for ADC_b=0,400_ (p < 0.001) and for ADC_b=0,1000_ (p = 0.02) the values between benign and malignant fractures differed significantly while for ADC_b=0,2000_ (p = 0.3) no significant difference was observed. Accordingly, for the differentiation of both entities, ADC_b=0,400_ achieved an AUC of 0.785 (accuracy = 0.773; sensitivity: 0.813; specificity: 0.735; cutoff: 1.265 × 10^–3^ mm^2^/s) followed by ADC_b=0,1000_ with an AUC of 0.698 (accuracy = 0.742; sensitivity: 0.906; specificity: 0.588; cutoff: 0.805 × 10^–3^ mm^2^/s) and lastly ADC_b=0,2000_ with an AUC of 0.592 (accuracy = 0.621; sensitivity: 0.844; specificity: 0.412; cutoff: 0.545 × 10^–3^ mm^2^/s).

#### 3.1.2 Signal Intensity Ratio (SIR)

In brief, for SIR_b=400_ (p < 0.001), SIR_b=1000_ (p < 0.001) and SIR_b=2000_ (p < 0.001)

significant differences between both groups were observed.

SIR_b=1000_ achieved the best discriminative performance to differentiate both entities (AUC = 0.919; accuracy = 0.909; sensitivity: 0.969; specificity: 0.853; cutoff: 1.497) followed closely by SIR_b=2000_ (AUC = 0.917; accuracy = 0.909; sensitivity: 0.906; specificity: 0.912; cutoff: 1.074) and lastly by SIR_b=400_ (AUC = 0.841; accuracy = 0.803; sensitivity: 0.938; specificity: 0.676; cutoff: 2.96). Importantly, for SIR_b=2000_ the optimal cutoff based on the Youden index was computed as very slightly above 1, which signifies the transition from an isointense to a hyperintense image impression.

#### 3.1.3 Qualitative analysis

In brief, except for the b = 2000 s/mm^2^ DWI trace images, there was a large overlap between the visual signal intensity characteristics of benign and malignant vertebral fractures as assessed qualitatively on ADC maps and DWI trace images. For the b = 2000 s/mm^2^ DWI trace images, a specificity of 0.794 and a sensitivity of 1 was achieved for the differentiation of benign from malignant vertebral fractures when relying on the presence of hyperintense signal as the cutoff.

### 3.2 Performance of combined metrics

All full ML models achieved a high diagnostic performance in differentiating benign from malignant vertebral fractures. For the Full_b=400_ model, a random forest (optimal hyperparameter values: mtry = 2, splitrule = extratrees, min.node.size = 7) achieved an AUC in ROC of 0.95/1 for the training/testing data set respectively.

For Full_b=1000_ a random forest (optimal hyperparameter values: mtry = 1, splitrule = gini, min.node.size = 2) achieved an AUC in ROC of 0.98/1 for the training/testing data set respectively. For Full_b=2000_ a random forest (optimal hyperparameter values: mtry = 2, splitrule = extratrees, min.node.size = 19) achieved an AUC in ROC of 0.98/1 for the training/testing data set respectively.

For the reduced models, only the Reduced_b=2000_ model achieved a comparable performance to the full models thus further corroborating the high diagnostic yield of qualitative image information as obtained from b = 2000s/mm^2^ DWI. Specifically for the Reduced_b=2000_ model a random forest (optimal hyperparameter values: mtry = 1, splitrule = extratrees, min.node.size = 2) achieved an AUC in ROC of 0.97/1 for the training/testing data set respectively.

The Reduced_b=1000_ and Reduced_b=400_ models achieved an AUC in ROC of 0.87/0.88 and 0.87/0.61 for the training/testing data set respectively. The base-line model achieved an AUC in ROC of 0.71/0.77 for the training/testing data set respectively thus confirming the overall added value of DWI for the differentiation of benign from malignant vertebral fractures.

## 4 Discussion

In this head-to-head comparison study, using a state-of-the-art SShot ZOOM SE-EPI DWI sequence, we compared the capability of low to ultra-high b-values to differentiate benign from malignant vertebral fractures at 1.5 T.

We showed that when considering metrics individually, quantitative (signal intensity ratio - SIR) and qualitative metrics (grading of signal intensity of fracture) derived from DWI trace images exhibit an improved discriminative performance compared to metrics derived from ADC maps. Incidentally, the SIRs as derived from b = 1000 and b = 2000 s/mm^2^ DWI trace images individually allowed for an excellent separation of these two entities with the AUC reaching 0.92. Importantly however, an excellent separation of these entities could also be achieved simply by analyzing the signal intensity of the fracture of interest on b = 2000 s/mm^2^ DWI trace images. Importantly, our data suggests that a hyperintense signal on b = 2000 s/mm^2^ DWI trace images is highly indicative of a malignant fracture.

The overall added value of DWI for the differentiation of benign from malignant vertebral fractures was confirmed by the baseline ML model (i.e. considering only qualitative metrics from STIR T2w, T1w and T2w imaging), that only achieved an AUC in ROC of 0.71/0.77 for the training/testing data set respectively. When combining all metrics (from conventional imaging and DWI), an excellent separation (AUC: 0.95-0.98 in training set) of both entities could be achieved irrespective of the choice of b-value. However, when relying solely on qualitative metrics, only the model relying on b = 2000 s/mm^2^ data could match the performance of the full models relying on qualitative and quantitative metrics. This further corroborates the high diagnostic yield of qualitative b = 2000 s/mm^2^ image analysis.

In a recent meta-analysis, the impact of the choice of b-value on the discriminative performance of individual DWI/ADC metrics for the differentiation of vertebral fractures was investigated [5]. The authors concluded that low-b-values (i.e. below 500 s/mm^2^) are superior to standard b-values (i.e. above 500 s/mm^2^) for the computation of ADC maps and thus for subsequent differentiation of the two entities. Accordingly, the differences in ADC values between benign and malignant fractures were greater on ADC maps derived from lower b-values than those derived from higher b-values. Furthermore, ADC values in general were found to be lower on ADC maps derived from higher b-values [5]. We confirm this finding in our study, as the diagnostic performance of the ADC also decreased with increasing b-values and ADC values decreased with increasing b-values. Notably, Park et al. [4] similarly observed reduced diagnostic performance in differentiating benign from malignant fractures by using ADC maps derived from higher b-values. Specifically, using a standard SShot SE-EPI DWI sequence, the authors achieved a sensitivity and specificity of 80.5 % and 76 % respectively for the ADC maps derived from b-values of 0 and 400 s/mm^2^ and a sensitivity and specificity of 63 % and 85 % respectively for ADC maps derived from b-values of 0 and 1000 s/mm^2^ [4]. In this regard it should be noted that at different b-values, different underlying effects may also impact the image information. For example, at higher b-values, non-Gaussian diffusion effects (i.e. diffusion kurtosis) may also be included in the image. Furthermore, even for tissues with a mono-exponential dependence on diffusion, the ADC value as computed from two b-values is known to be affected by the baseline SNR, the true tissue ADC, and the selected high b values. It has been shown that the increased baseline noise in the DWI image at high b values can lead to a systematic bias in estimating the signal reduction due to true diffusion. This may result in a lower measured ADC [25]. Such effects may also explain the differences in diagnostic accuracy at various b-values.

Furthermore, we would briefly like to address the benefits of our ZOOM SE-EPI DWI sequence. By using a tilted refocusing pulse to reduce the phase-encoding FOV, geometrical distortion, image blurring and aliasing can be minimized. Especially when imaging the spinal cord, this sequence poses considerable benefits over its non-ZOOM prepared counterparts. Thus, this sequence allowed us to acquire high b-value images robustly at an acceptable scan time [8]. In this regard it should also be noted that we increased the number of signal averages (NSA) from 2 and 8 (at b = 400 and 1000 s/mm^2^) to 12 at b = 2000 s/mm^2^ to counteract possible SNR limitations associated with high b-value acquisitions, Furthermore, it should be noted that this sequence can also be used for diffusion tensor imaging (DTI) [8,26]. Accordingly, promising results have been reported for its application in the imaging of pediatric spinal tumors. While not investigated in this study, DTI parameters may potentially also serve as biomarkers for the differentiation of benign and malignant vertebral fractures. In this regard, a previous pilot study has shown the potential of DTI to characterize osteoporotic vertebral fractures [27].

Concerning the metric SIR, we observed an opposite trend for the SIR as compared to the ADC values as the diagnostic performance of the SIR metric increased with higher b-values.

By using the SIR metric (individually) as a means of differentiating benign from vertebral fractures an excellent AUC of 0.92 could be achieved both for b = 1000 and b = 2000 s/mm^2^ DWI trace images. As indicated above, the AUC for b = 400 s/mm^2^ was lower (0.841), yet, interestingly, still higher than what could be achieved by using values derived from ADC maps.

Most importantly however, the optimal cutoff for SIR differed between b = 1000 and b = 2000 s/mm^2^. Specifically, for b = 1000 s/mm^2^ DWI trace images, an optimal cutoff of 1.497 was computed whereas for b = 2000 s/mm^2^, an optimal cutoff of 1.074 was found. This opens up the possibility of considerably simplifying the process of differentiating the two entities by using b = 2000 s/mm^2^ DWI trace images, as an SIR of close to 1 signifies the transition from an isointense to a hyperintense image impression.

This could be confirmed by grading the fractures qualitatively: By using a hyperintense image impression as a “threshold” on b = 2000 s/mm^2^ DWI trace images, a specificity of 0.794 and a sensitivity of 1 was achieved for the differentiation of benign from malignant vertebral fractures. Specifically, a hyperintense signal on b = 2000 s/mm^2^ DWI trace images was highly indicative of a malignant fracture.

Sung et al. [7] also observed an increase in the frequency of hyperintense signal in malignant fractures when switching from b = 800 to b = 1400 s/mm^2^ DWI trace images as acquired with a standard SShot SE-EPI sequence [7].

In contrast, the diagnostic yield of b = 1000 and b = 400 s/mm^2^ DWI trace images or ADC maps for qualitative grading was low, as the frequency of hypo-, iso- and hyperintense fractures was much more evenly distributed between the benign and malignant fracture groups.

Thus, by visually inspecting b = 2000 s/mm^2^ DWI trace images as acquired with a SShot ZOOM SE-EPI DWI sequence it seems that an accurate differentiation of benign and malignant vertebral body fractures can be achieved without having to resort to calculating quantitative metrics, as firstly a hyperintense signal was indicative of a malignant fracture and secondly an isointense signal was indicative of a benign fracture. This approach thus has the potential to considerably simplify and accelerate the diagnostic process.

A future prospective and dedicated study should investigate whether this finding can be reproduced in a larger cohort of patients.

By pooling the diagnostic information of different metrics, as in clinical routine, an even better diagnostic performance may be achieved. Our full models integrating qualitative and quantitative metrics from conventional imaging and DWI achieved a nearly perfect diagnostic performance of 0.95−0.98 (AUC) in differentiating benign from malignant fractures irrespective of the b-value. In contrast, the baseline model that only incorporates information from conventional imaging, only achieved a diagnostic performance of 0.71−0.77 (AUC) thus confirming the overall added value of DWI. However, and importantly, the reduced b = 2000 s/mm^2^ model incorporating solely qualitative image information (from conventional imaging and DWI) achieved an AUC of 0.97 thus matching the performance of the full models. This further corroborates the impressive stand-alone performance of the qualitative image information that can be gained from b = 2000 s/mm^2^ images.

Lastly, our study has certain limitations: Firstly, this was a single center study encompassing only data from two MR scanners from a single vendor and obtained at a single field strength. This is of relevance as, besides the choice of b-values, the ADC is also affected by the magnetic field strength of the MR scanner and the exact choice of the pulse sequence (amongst other factors). Furthermore, while comparable to that of other studies [3,23], our sample size was limited, and our study cohort was quite heterogenous. Specifically, the exact signal characteristics of various benign and malignant entities may differ considerably. This may have impacted our results. Also, concerning the limited sample size, it should be noted that larger datasets are likely to decrease the risk of overfitting the machine learning classifiers. To partially counteract this limitation, we implemented 5-fold cross validation of our results. Thirdly, readers may not have been fully blinded towards the diagnoses in all cases as certain patients presented with multiple (sometimes older) vertebral fractures, which may have given away the diagnoses. Fourthly, qualitative image analysis was performed in consensus. As there was a large difference in experience between the readers, the scores from consensus reading may mostly reflect the analysis of the senior expert radiologist, which can be considered a limitation. Lastly, we included all patients with benign or malignant vertebral fractures irrespective of their image appearance on conventional imaging as we sought to define the general diagnostic yield of qualitative and quantitative metrics from SShot ZOOM SE-EPI DWI. In other words, we did not focus specifically on fractures with an atypical appearance in conventional imaging. A future study specifically assessing the value of DWI for the differentiation of benign and malignant vertebral fractures with atypical appearance in conventional imaging may be of interest.

In conclusion, using quantitative and qualitative metrics from SShot ZOOM SE-EPI DWI, benign and malignant vertebral fractures can be differentiated with high diagnostic accuracy. Importantly qualitative analysis of ultra-high b-value images may suffice for differentiation as well.

## Ethics statement

This study was approved by the local Ethics Committee and conducted according to the principles of the Declaration of Helsinki. General written informed consent was obtained from all subjects.

## Funding

This research did not receive any specific grant from funding agencies in the public, commercial, or not-for-profit sectors. Financial support for open access publication fees was granted by University of Zürich, Switzerland.

## CRediT authorship contribution statement

**Elisabeth Sartoretti:** Conceptualization, Data curation, Investigation, Formal analysis, Methodology, Software, Visualization, Writing - original draft, Writing - review & editing. **Sabine Sartoretti-Schefer:** Conceptualization, Data curation, Investigation, Formal analysis, Methodology, Software, Visualization, Writing - original draft, Writing - review & editing. **Luuk van Smoorenburg:** Data curation, Investigation, Writing - review & editing. **Barbara Eichenberger:** Data curation, Investigation, Writing - review & editing. **Árpád Schwenk:** Conceptualization, Supervision, Validation, Visualization, Writing - original draft, Writing - review & editing. **David Czell:** Conceptualization, Supervision, Validation, Visualization, Writing - original draft, Writing - review & editing. **Alex Alfieri:** Conceptualization, Supervision, Validation, Visualization, Writing - original draft, Writing - review & editing. **Andreas Gutzeit:** Conceptualization, Supervision, Validation, Visualization, Writing - original draft, Writing - review & editing. **Manoj Mannil:** Conceptualization, Data curation, Investigation, Formal analysis, Methodology, Software, Visualization, Writing - original draft, Writing - review & editing. **Christoph A. Binkert:** Conceptualization, Supervision, Validation, Visualization, Writing - original draft, Writing - review & editing. **Michael Wyss:** Conceptualization, Data curation, Investigation, Formal analysis, Methodology, Software, Visualization, Writing - original draft, Writing - review & editing. **Thomas Sartoretti:** Conceptualization, Data curation, Investigation, Formal analysis, Methodology, Software, Visualization, Writing - original draft, Writing - review & editing.

## Declaration of Competing Interest

Michael Wyss is a part-time employee of Philips Healthcare. The other authors declare no conflict of interest in relation to this article.
